# Implementation and evaluation of a quality improvement initiative to reduce late gestation stillbirths in Australia: Safer Baby Bundle study protocol

**DOI:** 10.1186/s12884-020-03401-0

**Published:** 2020-11-13

**Authors:** C. J. Andrews, D. Ellwood, P. F. Middleton, A. Gordon, M. Nicholl, C. S. E. Homer, J. Morris, G. Gardener, M. Coory, M. Davies-Tuck, F. M. Boyle, E. Callander, A. Bauman, V. J. Flenady, on behalf of the Safer Baby Bundle collaborators

**Affiliations:** 1 Centre of Research Excellence in Stillbirth, Mater Research Institute, The University of Queensland, Mater Health Services, Level 3 Aubigny Place, South Brisbane, QLD 4101 Australia; 2 Gold Coast University Hospital, and School of Medicine, Griffith University, Gold Coast, Australia; 3 SAHMRI Women and Kids, South Australian Health and Medical Research Institute, Adelaide, Australia; 4 Charles Perkins Centre, University of Sydney, Sydney, NSW Australia; 5 University of Sydney, Sydney, NSW Australia; 6 Burnet Institute, Melbourne, Victoria Australia; 7 The Ritchie Centre, Department of Obstetrics and Gynaecology, Monash University, Clayton, VIC Australia; 8 Institute for Social Science Research, The University of Queensland, Brisbane, Australia; 9 School of Public Health and Preventive Medicine, Monash University, Melbourne, Australia

**Keywords:** Stillbirth, Maternity care, Care bundle, Protocol, Quality improvement, Implementation

## Abstract

**Background:**

In 2015, the stillbirth rate after 28 weeks (late gestation) in Australia was 35% higher than countries with the lowest rates globally. Reductions in late gestation stillbirth rates have steadily improved in Australia. However, to amplify and sustain reductions, more needs to be done to reduce practice variation and address sub-optimal care. Implementing bundles for maternity care improvement in the UK have been associated with a 20% reduction in stillbirth rates. A similar approach is underway in Australia; the Safer Baby Bundle (SBB) with five elements: 1) supporting women to stop smoking in pregnancy, 2) improving detection and management of fetal growth restriction, 3) raising awareness and improving care for women with decreased fetal movements, 4) improving awareness of maternal safe going-to-sleep position in late pregnancy, 5) improving decision making about the timing of birth for women with risk factors for stillbirth.

**Methods:**

This is a mixed-methods study of maternity services across three Australian states; Queensland, Victoria and New South Wales. The study includes evaluation of ‘targeted’ implementer sites (combined total approximately 113,000 births annually, 50% of births in these states) and monitoring of key outcomes state-wide across all maternity services. Progressive implementation over 2.5 years, managed by state Departments of Health, commenced from mid-2019. This study will determine the impact of implementing the SBB on maternity services and perinatal outcomes, specifically for reducing late gestation stillbirth. Comprehensive process, impact, and outcome evaluations will be conducted using routinely collected perinatal data, pre- and post- implementation surveys, clinical audits, focus group discussions and interviews. Evaluations explore the views and experiences of clinicians embedding the SBB into routine practice as well as women’s experience with care and the acceptability of the initiative.

**Discussion:**

This protocol describes the evaluation of the SBB initiative and will provide evidence for the value of a systematic, but pragmatic, approach to strategies to reduce the evidence-practice gaps across maternity services. We hypothesise successful implementation and uptake across three Australian states (amplified nationally) will be effective in reducing late gestation stillbirths to that of the best performing countries globally, equating to at least 150 lives saved annually.

**Trial registration:**

The Safer Baby Bundle Study was retrospectively registered on the ACTRN12619001777189 database, date assigned 16/12/2019

## Background

Stillbirth is a tragic event for the woman, her partner, family and friends, as well as the healthcare professionals involved. Stillbirth imparts significant costs across the health system and society [1]. Every day in Australia six babies are stillborn, amounting to more than 2000 deaths a year [2]. For Aboriginal and Torres Strait Islander and some migrant and refugee communities (South Asian [3] and African [4]), stillbirth rates are often doubled [5].

In 2015, the stillbirth rate after 28 weeks’ gestation in Australia was 35% higher than countries with the lowest rates globally [6]. The average annual rate reduction at that time was 1.4%, ranking Australia 15th across high-income developed countries. Since then reductions in late gestation stillbirth rates have steadily improved [7, 8]; however, more can be done to reduce stillbirths in line with comparable countries [6]. A co-ordinated national approach to reducing practice variation and addressing areas of sub-optimal care provision is needed for further reductions in stillbirth. High quality clinical audits [9] suggest around 20 – 30% of late gestation stillbirths could be avoided with better care [10].

Working in partnership with parents, healthcare professionals, professional colleges, parent advocacy organisations, and government agencies, the Centre of Research Excellence in Stillbirth (Stillbirth CRE) identified key evidence-practice gaps in stillbirth prevention [11]. In early 2018, the Australian Senate convened a committee to inquire and report on the future of stillbirth research and education in Australia. The committee’s report [12] made a number of key recommendations encompassing improving the quality of antenatal care to address stillbirth in Australia. In response the government provided additional funding to the Stillbirth CRE to support national rollout of the Safer Baby Bundle (SBB), recognizing this key initiative to ensure that pregnant women are provided with high quality, evidence-based antenatal care that reduces the risk of stillbirth [13].

Care bundles like the SBB are used frequently in health care with the aim of improving patient outcomes. They typically contain three to five evidence-based elements designed to formalise care and/or reduce practice variation [14]. Care bundles in stillbirth prevention in the UK have shown benefit [15, 16]. The Scottish Maternity and Children Quality Improvement Collaborative (MCQIC) has been associated with a stillbirth rate reduction of 22.5% since 2014 [16, 17] and the Saving Babies Lives Care Bundle (SBLCB) evaluation in England showed a 20% reduction [15]. The four care elements included in the SBLCB were: smoking monitoring and cessation strategies; monitoring fetal growth; reduced fetal movements; and effective fetal monitoring in labour [15].

### The Safer Baby Bundle

The SBB is modelled on the SBLCB [15] with modifications made through extensive consultation with the project’s partners and collaborators, national experts, professional bodies, parent advocates and a survey of lead clinicians across Australian maternity hospitals [18]. The survey highlighted gaps in care for each of the proposed elements. The SBB, which is founded on evidence-based recommendations for Australia and New Zealand [19–24], aims to address these gaps.

The five SBB elements address commonly identified evidence practice gaps: 1) supporting women to stop smoking in pregnancy, 2) improving detection and management of fetal growth restriction (FGR), 3) raising awareness and improving care for women with decreased fetal movements (DFM), 4) improving awareness of maternal safe going-to-sleep position in late pregnancy, 5) improving decision making about the timing of birth for women with risk factors for stillbirth. Specific recommendations for the five elements are detailed in the SBB Handbook and Resource Guide [25], which has been endorsed by peak professional bodies, parent advocacy organisations, Departments of Health partners, and was made publicly available in October 2019.

Implementation of the SBB is supported by a comprehensive package of evidence-based and collaboratively developed resources. These include: best practice recommendations; implementation tools including clinical checklists and management algorithms; key performance indicators and audit; an educational program for healthcare providers (eLearning and face-to-face training); information and educational resources for women and their families; and a communications and awareness campaign.

In January 2019, the Stillbirth CRE, in partnership with the Stillbirth Foundation Australia, Still Aware and Departments of Health across Queensland (QLD), Victoria (VIC) and New South Wales (NSW), received funding to implement and evaluate the SBB across these three states. Commencing from mid-2019, implementation of the SBB was undertaken in partnership with these three state Departments of Health. Commencement dates, implementation strategies and approaches vary across jurisdictions but the intent for all is to progressively embed the SBB within existing care over a 12-24month implementation period (post-launching).

Subsequent to the original development of this study, the SARS-COV-2 (COVID-19) was identified by the World Health Organization as a global health emergency and pandemic [26]. Isolation measures were first introduced in Australia in March 2020. Since then maternity care has changed due to restrictions imposed by the COVID-19 pandemic. For example, telehealth has replaced routine antenatal visits and women are reporting increased self-monitoring. At this time the overall impact of COVID-19 restrictions on implementation and evaluation of the SBB are uncertain. There is growing concern that stillbirth rates may increase during the pandemic as suggested by recent evidence from the UK [27]. The original protocol for this study has been amended here to extend the implementation time period to account for launch delays (for QLD) and disruptions to implementation activities, and to add measures to assess the indirect impact of COVID-19 restrictions on stillbirth and other important maternal and neonatal outcomes.

We report here the protocol for the evaluation of the SBB initiative and the state-led quality improvement programs supporting implementation across maternity services in NSW, QLD and VIC.

## Methods

### Study aims and objectives

The overall purpose of this study is to determine the impact of implementing the SBB on Australian maternity services and perinatal outcomes. The primary aim is to (1) demonstrate the effectiveness of the SBB in reducing late gestation stillbirth rates (28 weeks’ gestation or more). The study also aims to: (2) understand the processes and contextual factors influencing implementation success; (3) determine the impact of the SBB on change in awareness, knowledge, behaviours and experiences around providing or receiving antenatal care for healthcare professionals and women respectively; (4) to undertake an economic evaluation of the SBB.

The study’s primary objective is to compare stillbirth rates at 28 weeks’ gestation or more across Australian maternity settings pre and post implementation of the SBB. Secondary objectives include:

- Using routinely collected perinatal data to assess the endpoint effectiveness of the SBB for other important clinical outcomes, including any unintended consequences such as increased unnecessary intervention and preterm birth
- To explore variations in the provision of antenatal care influencing disparities in stillbirth rates for Aboriginal and Torres Strait Islander, migrant and refugee, and rural and remote women
- To evaluate the coverage, acceptability, feasibility, fidelity, and sustainability of the SBB initiative and its implementation across settings and for different stakeholders
- To explore indirect process and contextual factors influencing antenatal care provision in relation to the SBB elements due to COVID-19 pandemic restrictions through interviews and surveys with project leads
- To explore the views and experiences of women and maternity healthcare professionals with antenatal care in relation to the five SBB elements utilising surveys, focus groups and interviews
- To estimate the cost-effectiveness of the SBB compared to standard care.

### Study design

This study is a mixed methods, multi-centre, ‘before and after’ evaluation of the implementation of the SBB in three health jurisdictions. The study design is pragmatic to account for differences across jurisdictions for the level of implementation support provided and commencement times. VIC commenced implementation in June 2019, NSW launched in February 2020 and although QLD commenced baseline data collection from January 2020 their launch planned for March 2020 was delayed to October 2020 due to COVID-19. The study ‘implementation period’ (which includes pre and post implementation data collection as part of the implementation strategy) will therefore run for 2.5 years, followed by a 2 year post-implementation (maintenance phase). Data collection for final evaluation is anticipated to end by December 2023, with analysis and final reporting completed by June 2024. Given the uncertainties posed by the COVID-19 pandemic, there may be further delays in opportunities for data collection and implementation activities, however, this does not affect the framework for evaluation outlined here.

### Study setting

The SBB initiative, education program and all associated resources have been promoted and made accessible state-wide to all hospitals and healthcare professionals providing maternity care in NSW, VIC and QLD. The initial rollout of the SBB in these jurisdictions is managed through state-specific SBB implementation programs providing targeted support to hospitals recruited to and engaged with these programs. Thus, while all public and private maternity hospitals will be exposed to the SBB initiative through access to eLearning and resources, initially state-wide uptake and implementation will be driven by participation in formal quality improvement programs. These programs provide the opportunity for an in-depth evaluation of such approaches by facilitating collection of comprehensive process and impact data.

This study includes all public and private hospitals providing maternity care in NSW, VIC and QLD. Sites are grouped by level of implementation support into: (1) ‘targeted’ implementers, maternity hospitals recruited to and engaged with state-led SBB implementation programs; (2) ‘non-targeted’ implementers, all other maternity hospitals in each state. For ‘targeted’ implementers, participation was open to maternity hospitals across the three jurisdictions, with those who expressed an interest in joining recruited. Up to August 2020, 83 sites/hospitals were recruited (VIC- 23 sites across all 6 health regions, NSW- 25 sites across 4 Local Health Districts and QLD- 36 sites across 17 Hospital & Health Services). These sites account for approximately half of all births in these states.

Change in stillbirth rates and other important clinical outcomes will be described by state-wide routinely collected perinatal data for all births. Comparisons will be described by state, ‘targeted’ versus ‘non-targeted’ implementers, and between ‘targeted’ implementers. The study population includes women with a singleton pregnancy (28 weeks’ gestation or more) without lethal fetal anomalies attending for antenatal care.

For other aims and objectives, evaluations described here for process, impact, outcome and economic measures apply specifically to ‘targeted’ implementers (unless otherwise stated). The study population for these includes all women attending for antenatal care and healthcare professionals providing maternity care at ‘targeted’ implementer sites.

### Targeted implementation of the SBB through state-specific programs

State health departments of NSW, QLD and VIC will promote the SBB initiative across all maternity services in their jurisdictions, however, initial rollout involves targeted support to hospitals recruited to state-specific SBB implementation programs. Commencement dates, recruitment strategies and implementation approaches differ across jurisdictions but the intent for all is to progressively embed the SBB within existing care over a 12-24 month implementation period (post-launching). Each jurisdiction nominated a division (within their health department) to oversee the implementation process and data collection as follows: Safer Care Victoria (SCV, VIC Government), Clinical Excellence Commission (CEC, NSW Government) and Clinical Excellence Queensland (CEQ, QLD Government). In their capacity as state health care quality and safety improvement agencies, they are responsible for recruitment of ‘targeted’ implementers, implementation of the SBB in these sites and data collection for the purposes of the process and impact evaluations.

A key strategy used to optimise uptake of the SBB is having a dedicated implementation (quality improvement) project team for each jurisdiction, led by health service executive leadership teams. These teams will provide support through to the end of the project with the aim to see changes incorporated into business as usual. They will provide leadership, generate and sustain motivation for change, provide tools to support practice change through education, audit and feedback, and benchmarking and implementation support forums to facilitate sharing of experiences of the SBB by clinical champions from across ‘targeted’ implementers. In each state, at least three implementation learning support forums will be conducted during the implementation period, bringing together local implementation teams to discuss successes and challenges to enhance implementation. The Stillbirth CRE study co-ordination team will provide wide-ranging support across the jurisdictional implementation programs through attendance and participation at learning forums and representation on project teams and high-level committees. During both implementation and maintenance phases of this study, the Stillbirth CRE will report annual benchmarking of selected indicators and support national forums to share learnings.

The SBB educational program covers each element of the bundle and includes both face-to-face skills development and eLearning. The first phase of the educational resources for healthcare professionals (eLearning) was officially launched in October 2019, by the Australian Federal Minister for Health. Complementary information and educational resources for women and their families have also been developed. These will further support implementation of the SBB in a way that meets women’s needs and include a public awareness campaign. The SBB Education Working Group, with representation from all major parent advocacy organisations and professional colleges, will provide guidance on the integration of the awareness campaign [28] with the state-led SBB implementation strategy in the antenatal clinic setting.

### Evaluation design

**Fig. 1:**
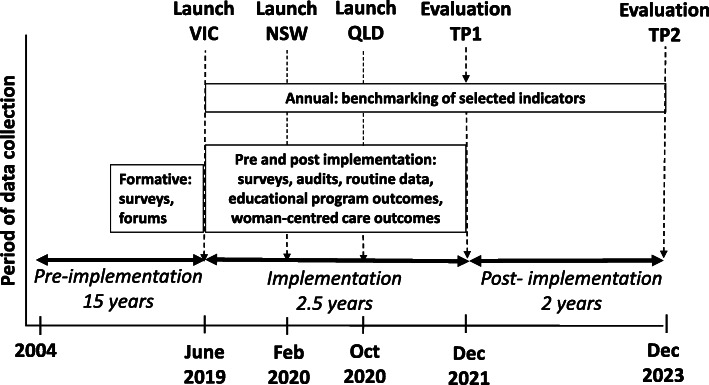
Study timeline and design. TP1- time point 1 (end of implementation period), TP2- time point 2 (2 years post-implementation)

A mixed-methods approach will be used to assess the processes, impacts and outcomes for the SBB initiative. Change in rates for the primary clinical outcome, stillbirth rate at 28 or more weeks, will compare the pre-implementation rate (including longer term trends over 15 years) with the rate in the 2 year post-implementation period (Time Point 2 (TP2)), using routinely collected perinatal data (see Fig. 1.) To allow for differences in commencement dates between jurisdictions and implementation disruptions due to COVID-19 restrictions, a 2.5 year implementation period is proposed. Key process and impact measures will be compared pre- (baseline) and post-implementation at evaluation TP1 including data from surveys of women and healthcare professionals, clinical audits and implementation process data. A survey of maternity service leads will be undertaken at TP1 (post-implementation) to assess changes in maternity services resource use, level of implementation and the influence of leadership, governance and workforce culture on implementation.

### Evaluation measures

**Fig. 2:**
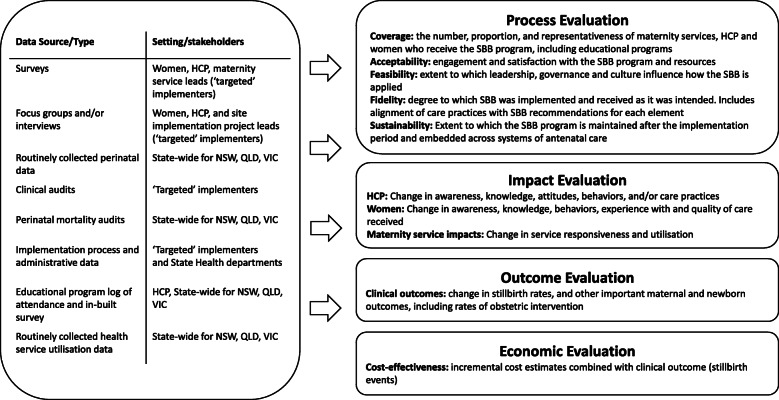
Data collection tools used in the SBB program evaluation. This brings together data collected across different settings and stakeholders and indicates how these will contribute to process, impact, outcome, and economic evaluations. HCP- Healthcare professionals, NSW- New South Wales, QLD- Queensland, VIC- Victoria, SBB- Safer Baby Bundle

We will use a framework commonly applied to complex public health service research [29], adapted to suit this clinical context. This framework integrates process, impact, outcome and economic evaluations across different settings and stakeholder levels including: State level health regions, maternity service providers/hospitals, quality improvement leadership teams, maternity healthcare professionals and women receiving antenatal care. Both quantitative and qualitative methods will be used to collect measures across these settings (Fig. 2).

An adaptive evaluation approach to address the additional complexity of the impact of the COVID-19 pandemic on implementation and evaluation of the SBB is required. Evaluation measures and data collection tools may need amending in response to changing needs, priorities, constraints, and opportunities. Within the existing framework, the indirect impact of the COVID- 19 pandemic on SBB implementation across process, impact, outcome, and economic evaluations will need to be considered.

**Process measures** collected will assess the coverage, acceptability, feasibility, fidelity, and sustainability of the SBB program and its implementation at ‘targeted’ implementors across settings and for different stakeholders. These measures include; healthcare professionals experience and satisfaction with resources including completion rate of educational programs; women’s experience including acceptability of information materials and satisfaction with care; and for each element key measures indicating alignment of antenatal care practices with the SBB recommendations, Table 1.

**Table 1:** Planned evaluation measures relating to the five elements of the Safer Baby Bundle

Element	Level of evaluation	Measure
Element 1: supporting women to stop smoking in pregnancy	Process	Proportion of women who are asked about their smoking status at first antenatal care visit and at 28 weeks antenatal appointment.
		Proportion of women who undertake exhaled breath carbon monoxide analysis at first antenatal care visit and at 28 weeks antenatal appointment.
		Proportion of women, identified as smoking or recent quitters at first antenatal care visit, who are provided with advice on the benefits of quitting.
		Proportion of women, identified as smoking, with documented referral to smoking cessation service (e.g. Quitline).
	Impact	Proportion of women, identified as smoking, with documented referral to smoking service who engaged with a smoking cessation service.
		Percentage of women who cease smoking between first antenatal care visit and birth
Element 2: improving detection and management of FGR	Process	Proportion of women with documented risk assessment for FGR at first antenatal care visit.
		Proportion of women (at any gestation) identified as at risk of FGR whose care was escalated as per the FGR care pathway^a^.
		Proportion of women with SFH measurement taken and plotted on growth chart at each antenatal visit from 24 weeks’ gestation.
		Proportion of stillbirths from 28 weeks’ gestation where substandard care for FGR detection or management are identified
	Maternity services impact	Proportion of term births with undetected FGR defined as severely growth restricted singletons (less than 3rd centile) undelivered at 40 weeks’ gestation (missed FGR)
	Outcome	Proportion of singleton babies delivered for suspected FGR at 37 weeks’ gestation or more who have a birthweight >25th centile.
Element 3: raising awareness and improving care for women with DFM	Process	Proportion of women provided with DFM information by 28 weeks’ gestation.
	Maternity services impact	Proportion of women with singleton pregnancies who have a CTG commenced within 2 hours of presenting (in person) at the maternity service with DFM, from 28 weeks’ gestation.
		Proportion of stillbirths from 28 weeks’ gestation where substandard care for DFM reporting or management are identified
		Percentage of women at 28 weeks’ gestation or more who attend a maternity service within 12hrs of DFM concern.
		Proportion of women with singleton pregnancies who present with DFM who undergo induction of labour or elective caesarean section before 39 weeks’ gestation for DFM as the only indication.
Element 4: improving awareness of maternal safe going-to-sleep position in late pregnancy	Process	Proportion of women who, by 28 weeks’ gestation, were given the information brochure on safe going-to-sleep position in late pregnancy.
	Impact	Proportion of women who report safe sleep practices after 28 weeks’ gestation.
		Proportion of women after 28 weeks’ gestation who can describe safe sleep practices (going to sleep on their side).
Element 5: improving decision making about the timing of birth for women with risk factors for stillbirth	Process	Proportion of women assessed for stillbirth risk factors at first antenatal care visit
		Proportion of women reassessed for stillbirth risk factors at 34-36+6 weeks’ gestation
	Impact	Proportion of women who report being involved as much as they wanted in decision-making about timing of birth
	Outcome	Proportion of women with singleton pregnancies who undergo induction of labour or elective caesarean section before 39 weeks’ gestation.

Abbreviations: *FGR* Fetal growth restriction, *SFH* Symphyseal-fundal height, *DFM* Decreased fetal movements, *CTG* Cardiotocography

^a^PSANZ/Stillbirth CRE FGR care pathway for singleton pregnancies [21]

**Impact measures** will assess the SBB programs effect on participants and include; healthcare professionals reported change in awareness, knowledge, attitudes, behaviours, and/or practices with implementation of the SBB and more broadly through use of the educational program; women’s reported change in awareness and behaviours in response to the five elements of care, as well as changes in experience with and quality of care received; maternity service reported change in service responsiveness and utilisation, Table 1.

**Outcome measures** focus on the endpoint effectiveness of the SBB program and demonstrate changes in clinical outcomes. The **primary clinical outcome** is stillbirth at 28 weeks’ or more gestation in singleton pregnancies without lethal fetal congenital anomalies. Secondary clinical outcomes will be collected from routinely collected perinatal data and include important maternal and new-born outcomes (including rates of obstetric intervention), Table 2.

**Table 2:** Key clinical outcomes

Clinical Outcomes	Measure	
Primary outcome	Stillbirth at 28 weeks’ or more gestation in singleton pregnancies without lethal fetal congenital anomalies	
Secondary outcomes	Fetal/Neonatal	Stillbirth; 20 weeks’ or more gestation; 28 weeks’ or more gestation; 37 or more weeks’ gestation; associated with substandard care factors (undetected FGR, poor DFM reporting or management); cause specific (PSANZ classification^a^)
		Neonatal death; early (within 7 days of birth) or late (within 7-28 days of birth)
		Neonatal hypoxic ischaemic encephalopathy (mild, moderate, or severe grading- ANZNN criteria^b^)
		Small for gestational age; birthweight < 10^th^ centile; birthweight < 3^rd^ centile
		Neonatal seizures
		Preterm birth; early (birth before 32 weeks) or late (birth before 37 weeks)
		Early term birth (birth 37-38 weeks)
		Admission to nursery; special care and/or intensive care; length of stay
		Need for respiratory support (defined using ANZNN criteria^b^)
		Early and late onset neonatal infection (defined using ANZNN criteria^b^)
	Maternal	Induction of labour
		Caesarean section; elective; emergency (caesarean section birth after labour)
		Admission to intensive care
		Unplanned returned to theatre

^a^Perinatal Society of Australia and New Zealand (PSANZ) classification [30], ^b^Australian and New Zealand Neonatal Network (ANZNN) criteria [31]

**Economic measures** will evaluate incremental cost-effectiveness of the SBB compared to standard care, total and incremental costs to the health system, and total and incremental costs to society. Measures include all inpatient and outpatient health service utilisation from first antenatal booking to six weeks postpartum including antenatal ultrasound scans and maternal and neonatal length of stay from routinely collected administrative data sources.

### Data collection and management

**Fig. 3:**
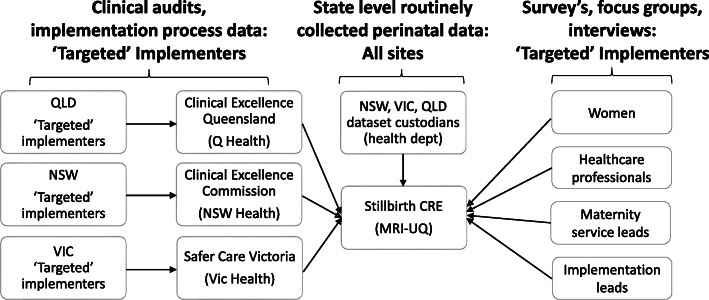
Data collection processes

The nominated division within state health departments (CEC, CEQ and SCV) will be responsible for data collection from ‘targeted’ implementers. Each state has identified a data custodian to oversee this data collection and management. The study coordinating centre, Stillbirth CRE, will be responsible for the overarching evaluation and management of data provided to them from the health department (Fig. 3.). Investigators from the study coordinating centre will conduct surveys, focus groups, and one-to-one interviews with women, healthcare professionals, maternity service leads and implementation project leads.

#### Non-identifiable electronic extract of routinely collected perinatal data

Approval will be sought through the relevant state perinatal data collection custodians to access and analyse routinely collected population-based surveillance system data covering all births in NSW, VIC and QLD public and private hospitals over the study time period (covering 15 years’ pre-implementation to 2 years’ post-implementation). This non-identifiable electronic data will be extracted from each of the participating jurisdictions at yearly intervals throughout the duration of the project.

Monitoring and timely reporting of any unintended harm as a result of SBB implementation, such as increased unnecessary intervention and preterm birth, is essential [32]. Annual analysis of routine data extracts throughout both implementation and maintenance phases will allow for benchmarking of selected indicators. Reporting of comparisons for stillbirth rates and other important clinical outcomes will be for both ‘targeted’ and ‘non-targeted’ implementers. These comparisons will describe the effect of state-wide uptake of SBB components over time and consider any indirect impacts on antenatal care provision due to COVID-19 restrictions by jurisdiction.

Requested data items include: maternal demographics and medical history; previous pregnancy complications and birth outcomes; current pregnancy details; labour and birth outcomes; neonatal outcomes; and hospital demographics. Routinely collected hospital service utilisation data for each participating hospital will also be obtained as part of the SBB dataset. These data are routinely collected and reported to state health departments as part of minimum reporting requirements for activity-based funding.

#### Clinical audits and implementation process data

Jurisdictions may undertake and report a series of audits to assess process evaluation measures including missed cases of FGR, reporting of DFM and women birthing at term (measures such as risk assessment and management according to risk, smoking cessation support). Through these audit and feedback, benchmarking of key performance indicators will flag priority areas for practice change. ‘Targeted’ implementers will report on a standard set of process, impact and outcome measures for each of the five elements as part of their quality improvement strategies to implement. This data is important to understand the fidelity of the implementation strategies and sites will provide these implementation process data through the respective health departments (CED, CEC, SCV).

High quality clinical audit of all stillbirths to identify substandard care factors will be promoted through provision of the IMproving Perinatal Mortality Review and Outcomes Via Education (IMPROVE) program [33]. The data collection tool recommended by PSANZ [30] will be recommended for use across all participating maternity services to inform the standard clinical review of all stillbirths.

#### Surveys

Surveys will be undertaken at ‘targeted’ implementing sites. Surveys of women, healthcare professionals and maternity service leads are based on those used in the SBLCB evaluation [15] and will be administered electronically pre and post implementation (TP1). The self-administered surveys will be undertaken using an online survey software tool, Checkbox (Checkbox Survey Inc., Watertown, MA, USA) or REDCap [34]. Survey responses will be extracted from the on-line survey tool and imported into IBM SPSS statistics 24.0 for analysis.

##### Survey of women

This survey around care practices has been adapted from the UK SBLBC [15], in consultation with parents, clinicians and Aboriginal and Torres Strait Islander representatives on the investigator team, to include questions relevant to the SBB elements and to incorporate relevant International Consortium for Health Outcomes Measurement (ICHOM) data items for pregnancy and child birth [35]. Survey questions (n = 69) address demographic characteristics, pregnancy and birth outcomes, and experience of care received around the relevant elements of care. The survey questions are largely multiple choice with an option to provide comment. The survey will be administered to women following the birth (before hospital discharge or within 6 weeks of birth). Women who had a stillbirth or neonatal death prior to discharge from the hospital of birth will be excluded. Healthcare professionals will identify and approach all eligible women after the birth providing written materials along with a verbal explanation of the SBB study. Women will complete a self-administered on-line survey using a personal electronic device (i.e. phone, tablet, laptop) or electronic tablet provided by the study. By completing and submitting the survey, the women will be indicating consent to participate. Women will be reassured that nonparticipation or withdrawal from the study will not affect their routine care, relationships with professional staff or ongoing relationship with their health services provider.

##### Survey of healthcare professionals

Healthcare professionals providing antenatal care at ‘targeted’ implementing sites will be invited to complete an anonymous questionnaire to elicit attitudes, knowledge and practices around the SBB, pre- and post-implementation. The survey draws on the UK SBLBC [15] and consists of 50 questions and is constructed around 3 domains: demographics of clinicians (n = 10); clinicians current experience with care practices around SBB for each element (n = 38); clinicians views about service responsiveness (n = 2). For each element of the SBB there are questions around; the frequency of best practice for key recommendations; guideline availability; adequacy of training; satisfaction with resources; and clinician’s attitudes towards having conversations with women about these elements of care. There is consistency with our previous survey [18] of Australian maternity services in the rating scales for best practice frequency and questions relating to guidelines.

Eligible healthcare professionals will be identified and recruited by members of the respective site implementation teams. An e-mail invitation to participate in the survey will be circulated to all healthcare professionals providing antenatal care and will include a link to the on-line survey. The survey may also be distributed (on behalf of the Stillbirth CRE) by professional colleges including (but not limited to) the Australian College of Midwives, Women’s Healthcare Australasia and the Royal Australian and New Zealand College of Obstetricians and Gynaecologists. This would increase the reach of the survey to include healthcare professionals across ‘non-targeted’ implementers. By completing the survey consent to participate is implied. Participant information provided outlines that participation is voluntary, and that nonparticipation will not affect workplace relations.

##### Survey of maternity services leads

Based on that used in the UK SBLBC [15], this survey will be undertaken at ‘targeted implementer’ sites to examine the perceived level of implementation, and the impact on leadership, governance and workforce culture on implementation and change in key indicators and clinical outcomes post-implementation (TP1). This survey will be finalised during the implementation period drawing on the specific implementation approaches across each jurisdiction.

Maternity service leads and/or implementation leads will be invited to complete a self-administered on-line survey. The list of eligible leads and the contact for each will be identified by the state project teams. A brief description of the study together with an invitation to participate and a link to the online questionnaire will be provided in an email invitation.

#### Focus groups and semi-structured interviews

At ‘targeted’ implementer sites qualitative data will be collected to gain a more nuanced picture of women’s, healthcare professionals and implementation project leads experiences of the SBB by providing insights that may not easily be captured in quantitative data. These include issues related to acceptability of the various components of the SBB as well as important questions about possible differences in implementation, uptake and effects. Purposive sampling of interview participants will enable key dimensions of interest to the study and its outcomes to be explored in detail. Semi-structured interviews or focus groups will enable pre-determined questions to be explored while also inviting discussion of issues that are of importance to participants. All interviews will be conducted by a skilled qualitative interviewer, digitally recorded and transcribed in full for thematic analysis. Interviews will take place either in-person or by phone or videoconference. Women’s partners will also be invited to participate where possible.

For recruitment of women (and their partners) to focus groups/interviews, a member of the research team will liaise with clinical staff to identify those eligible who will then be approached directly by the site research team. Additionally, women and healthcare professionals who complete surveys are asked to provide their details (separately) if they consent to being contacted for follow up research related to this project. Women will be reimbursed for time spent participating in these activities and reimbursed for reasonable out-of-pocket expense such as child care and parking. Healthcare professionals will be approached directly by the site investigator and invited to participate. Implementation project leads will be approached by investigators from the study coordinating team. Evaluation through surveys and one-to-one interviews with ‘targeted’ implementer project team leads will further explore site recruitment strategies, commitment, and engagement to the SBB initiative and the influence of leadership, governance and culture on implementation strategies and approach.

#### Educational programs evaluation

Log of attendance and/or completion will be collected via the on-line registration platform for educational programs for both eLearning and face-to-face. Evaluation of participant knowledge and confidence around the five elements of the SBB will be undertaken using an in-built on-line survey administered immediately before and after completion of the eLearning module.

#### Economic evaluation

A modelled health economic evaluation will be undertaken. The intervention costs will be obtained from the routinely collected health service utilisation data. In addition, health service and societal costs of stillbirth will be captured using publicly available data sources or data from peer-reviewed publications for women who have a stillbirth and those who do not, developed as a part of the Stillbirth CRE [1]. In partnership with the Stillbirth CRE the economic evaluation is being conducted by Dr Emily Callander from Monash University. The detailed protocol developed for this component of the evaluation will be published separately and is beyond the scope of this paper.

### Sample size and data analysis

#### Clinical outcomes

Based on the most recent 3 year period prior to the SBB rollout, for which data are currently available (2016-2018), the late gestation stillbirth rate in Australia was 2.4 per 1000 births (unpublished data Stillbirth CRE). This decreased from 2.7 per 1000 births in 2013-2015 [6]. The SBB initiative aims to amplify and sustain a further rate reduction of at least 20% to target a rate of 1.9 per 1000 births by the end of 2023. Maintaining a downward pressure on rates is challenging given that as rates get lower further reductions may become increasingly more difficult to attain.

Based on 113,000 births per year across ‘targeted’ implementers, the study will be able to estimate a stillbirth rate of 1.9 to a precision of 1.7 to 2.1, with 95% confidence: 430 stillbirths out of 113,000 x 2 =1.9 per 1000 births. Put another way, the project will have 95% power (alpha=5%, two-sided) to show that an achieved stillbirth rate of 1.9 per 1000 births (the target for 2022-2023) is statistically significantly different from a stillbirth rate of 2.4 per 1000 births (pre-implementation for 2016-2018).

Secondary endpoints including obstetric intervention and other important maternal and newborn outcomes measurable using routine data will be analysed using analysis, similar to the pre-specified primary endpoint. Formal power calculations for the secondary endpoints have not been done due to statistical multiplicity. Point estimates for these secondary endpoints will be checked to confirm they are consistent with the result for the primary endpoint. Any outlying results will be investigated. Descriptive statistics will be used to summarise data such as demographic characteristics.

#### Survey data

The sample size for the survey of women is determined by the need to focus on a fixed period to encourage site compliance with a short-focussed collection period. A collection period of two- three weeks pre- and post-implementation across at least 30 sites each with approximately 2000 births per year will yield a total sample of approximately 2300 with a 50% completion rate (1150 in each period). This will provide approximately 500 women with DFM, 500 women who smoked at booking, and around 600 women with risk factors at term.

The sample size for the survey of healthcare professionals is based on two full time equivalents per 1000 births across all ‘targeted’ implementer sites and 60% availability and a 30% completion rate over the two-week survey period gives a total sample size of 6000 (3000 in each period pre and post implementation).

#### Focus groups and interviews

Thematic analysis [36] will be applied to the qualitative data collected throughout the study. NVivo software will be used to manage the qualitative data and to facilitate coding and development of themes. At least two researchers will read transcripts and independently establish coding categories before using an iterative approach to develop agreed themes and subthemes, with attention to contrasts across groups. Concordance across coders will be reviewed and discrepancies will be discussed to ensure conceptual consistency. Throughout the analysis, themes will be reviewed and discussed with members of the wider research team to include clinician, non-clinician and consumer perspectives and to strengthen the credibility and trustworthiness of findings.

#### Subgroup analyses

Where sample sizes allow, subgroup analyses by demographic characteristics of the maternity services and level of implementation (for ‘targeted’ implementers, as measured by process indicators and results of the leadership survey) will be undertaken for quantitative clinical endpoints and survey data. Maternity services characteristics to be explored include state, remoteness, private versus public, and service capability level according to the Australian Maternity Services Capability Framework [37]. Subgroup analysis will also explore variations in the provision of antenatal care and disparities in stillbirth rates and other clinical outcomes for Aboriginal and Torres Strait Islander, migrant and refugee, and rural and remote women. Subgroup analysis from the survey of women will be undertaken by parity, maternal age, and pregnancy complications. Subgroup analysis of the healthcare professional survey data will explore the influence of discipline (e.g. midwives, obstetricians).

### Data storage, access and archive

To ensure confidentiality, data will be stored securely on a password protected web-based database with access restricted to the research team only. Electronic files will be stored on secure password protected drives on the Mater Research Institute (MRI) network. Any associated paper copies of documentation will be kept in a locked secure environment by the Chief Investigator at MRI, Brisbane.

Data will be kept for a period of 7 years after the conclusion of the study, in accordance with institutional policies. The Mater Research Ownership, Storage and Retention of Human Research Materials and Data Policy (PY-RSH_300300) requires that permission from the Head of Department where the Chief investigator is employed should be obtained before moving or destroying the research data once the primary period of retention has finished.

### SBB study committees and advisory groups

An SBB Steering committee, made up of Stillbirth CRE representatives, leads from each jurisdictional partner organisation and a parent representative will meet quarterly to provide high level oversight for the SBB program. An operational committee, made up of the study chief, associate and partner investigators, and jurisdictional implementation team representatives will meet regularly to ensure successful implementation of the SBB. Overarching advice and support specific to consultation with Aboriginal and Torres Strait Islander, and migrant and refugee women (and their care providers) will be sought through the Stillbirth CRE Aboriginal and Torres Strait Islander and Migrant and Refugee Advisory Groups. The Stillbirth CRE Aboriginal and Torres Strait Islander Advisory Group follow the Guidelines for Ethical Research in Australian Indigenous Studies (Australian Institute of Aboriginal and Torres Strait Islander Studies) and relevant NHMRC ethical guidelines for research with Aboriginal and Torres Strait Islander Peoples [38].

### Dissemination plan

The final data analysis examining the change in stillbirth rates will commence once all the PDC data for each participating state has been submitted (Dec 2023). Some preliminary analysis of historical data (15 years pre-implementation) will begin once appropriate approvals are obtained and results will be presented at SBB dissemination events held annually (Stillbirth CRE Annual National Forum). These forums are open to maternity healthcare professionals, consumer representatives, policy makers and researchers from around the country to ensure wide audience is captured. Annual forums will also provide a platform for the Stillbirth CRE and Department of Health partners to report benchmarking for selected indicators throughout the study. Following data analysis, a comprehensive national report will be drafted which summarises the aggregate data. High-level aggregate data will be presented, and individual services will not be identifiable. In addition to clinical outcomes, this report will contain aggregate data from ‘targeted’ implementers for key process and impact measures relating to each element of the SBB. The final evaluation report is due to be submitted by July 2024.

Results of the study will also be submitted to peer reviewed journals for publication, presented at leading national and international professional conferences and further disseminated to the public and healthcare community, to raise awareness and inform best clinical practice in the prevention of stillbirth.

## Discussion

Stillbirth is a devastating pregnancy outcome resulting in profound and often long-lasting adverse psychosocial effects for the mother, father and family. Late gestation stillbith rates in Australia are unacceptably high. Through the Stillbirth CRE, we have established a critical mass of world-renowned experts in stillbirth prevention. Working in partnership with parents, we are in a unique position to significantly reduce the incidence of stillbirth. We have identified key evidence practice gaps in maternity care in stillbirth prevention and there is strong evidence on how to overcome these in several key areas. The challenge now is to translate the evidence into practice, and this is what the SBB for Australia will do.

Partnership with parent support and advocate organisations (Stillbirth Foundation Australia, Still Aware, and others) will ensure the voices of parents are heard through engaging parents and the community in development of materials and implementation strategies.

Our partnering state health department organisations share the Stillbirth CRE’s strong desire to reduce stillbirth rates and other adverse pregnancy outcomes by reducing gaps between evidence and practice in maternity care. These partners are the peak authorities for leading quality improvement in maternity health care for their jurisdictions. They formulate and disseminate policies that have a significant impact on health care services and delivery and are at the forefront of translation of health and policy practice. They will actively promote and support the SBB rollout across all maternity services in their jurisdictions, shaping the care practices recommended into policy. Initial rollout involves targeted support to those maternity hospitals recruited to each states SBB implementation programs. An in depth understanding of the factors which underpin successful implementation of the SBB in these services will guide future implementation activities and refinement of the SBB. As will insights into the added value of differing approaches to support implementation.

The Stillbirth CRE is working to upscale the SBB initiative across Australia and partnering with health departments of all remaining jurisdictions to commence implementing the SBB over 2020 to 2021. Implementation and evaluation across these other states/territories will vary and will be incorporated into a future planned national evaluation. We anticipate the SBB initiative will save the lives of over 150 babies each year if up scaled across all Australian jurisdictions. The findings of this study will also provide evidence for the value of a systematic, while pragmatic, approach to strategies to reduce the evidence-practice gaps across maternity services.

## Data Availability

The datasets used during the current study are available from the corresponding author upon reasonable request.

## Abbreviations

CEC: Clinical Excellence Commission
CEQ: Clinical Excellence Queensland
CTG: Cardiotocography
DFM: Decreased fetal movements
FGR: Fetal growth restriction
ICHOM: International Consortium for Health Outcomes Measurement
MCQIC: The Scottish Maternity and Children Quality Improvement Collaborative
NHMRC: National Health and Medical Research Council
NSW: New South Wales
PSANZ: Perinatal Society of Australia and New Zealand
QLD: Queensland
SBB: Safer Baby Bundle
SBLCB: Saving Babies Lives Care Bundle
SCV: Safer Care Victoria
SFH: Symphyseal-fundal height
Stillbirth CRE: The Centre of Research Excellence in Stillbirth
UK: United Kingdom
VIC: Victoria
